# A Review on Natural Fiber-Reinforced Geopolymer and Cement-Based Composites

**DOI:** 10.3390/ma13204603

**Published:** 2020-10-16

**Authors:** Marfa Molano Camargo, Eyerusalem Adefrs Taye, Judith A. Roether, Daniel Tilahun Redda, Aldo R. Boccaccini

**Affiliations:** 1 Institute of Biomaterials, Department of Materials Science and Engineering, University of Erlangen-Nuremberg, 91058 Erlangen, Germany; marfa.carolina.molano@fau.de (M.M.C.); eyerusalem.taye@fau.de (E.A.T.); 2 Institute of Technology, School of Mechanical and Industrial Engineering, Addis Ababa University, Addis Ababa 7754, Ethiopia; daniel.tilahun@aait.edu.et; 3 Institute of Polymer Materials, Department of Materials Science and Engineering, University of Erlangen-Nuremberg, 91058 Erlangen, Germany; judith.roether@fau.de

**Keywords:** geopolymers, fibers, composites, interphases, surface treatments

## Abstract

The use of ecological materials for building and industrial applications contributes to minimizing the environmental impact of new technologies. In this context, the cement and geopolymer sectors are considering natural fibers as sustainable reinforcement for developing composites. Natural fibers are renewable, biodegradable, and non-toxic, and they exhibit attractive mechanical properties in comparison with their synthetic fiber counterparts. However, their hydrophilic character makes them vulnerable to high volumes of moisture absorption, thus conferring poor wetting with the matrix and weakening the fiber–matrix interface. Therefore, modification and functionalization strategies for natural fibers to tailor interface properties and to improve the durability and mechanical behavior of cement and geopolymer-based composites become highly important. This paper presents a review of the physical, chemical and biological pre-treatments that have been performed on natural fibers, their results and effects on the fiber–matrix interface of cement and geopolymer composites. In addition, the degradation mechanisms of natural fibers used in such composites are discussed. This review finalizes with concluding remarks and recommendations to be addressed through further in-depth studies in the field.

## 1. Introduction 

The need for energy-efficient construction and industrial materials has stimulated the development of composites incorporating natural fibers and more environmentally friendly matrices. Significant benefits resulting from the use of natural fibers have been reported in the literature [1,2], including biodegradability, renewability, low density, relatively high specific strength properties, reduced tool wear (less abrasive to processing equipment), and low cost. On the other hand, geopolymers are gaining increasing interest as they can be produced at low temperature and made from industrial wastes such as fly ash (by-product from coal-fired power stations) and inorganic aluminosilicate materials such as metakaolin (calcined kaolin clay) [3]. Geopolymers are basically synthesized from aluminosilicate-based sources and alkaline activators mixed with water to be used in a wide range of applications [4]. In this context, natural fibers have been proposed as an environmentally attractive alternative to other (synthetic) reinforcement elements to develop geopolymer composites. In this regard, the literature has reported improved compression strength in fly ash-based geopolymers reinforced with untreated cotton, sisal, and coir fibers [5,6]. Kriven et al. [7,8] have also reported enhanced tensile and flexural strength in metakaolin-based geopolymers reinforced with alkali-treated bamboo fibers and alkali-treated jute weave when compared to untreated composites. Furthermore, the imprints observed of these fibers on the geopolymer matrix suggested an enhanced interfacial bonding provided by the treatment. Additionally, researchers are motivated to develop alternative approaches to reduce the carbon footprint in the production of cement composites by reinforcing them with natural fibers [9,10,11]. However, the main drawback of natural fibers to be used in cement and geopolymer composites is the high content of polarized hydroxyl groups derived from the lignin–hemicellulose compound, resulting in poor resistance to moisture absorption, low dimensional stability (shrinkage and swelling), and low fire resistance capacity [3,12]. Therefore, several studies [10,11,13,14,15] have focused on evaluating strategies to improve natural fiber performance and compatibility with cementitious and geopolymer matrices by tailored surface modification of fibers.

The number of approaches put forward and the different outcomes have prompted us to prepare the present review paper, given the importance of fiber treatments for the success of composite technology. The paper is therefore not intended as a comprehensive review of all natural fiber-reinforced cement-based or geopolymer composites developed to date. The paper presents rather a review of the effects that pre-treatments of natural fibers exert on the interface of cement and geopolymer-based composites. The content is organized in five principal sections as follows: (i) general outline of the microstructure, chemical composition and properties of natural fibers; (ii) description of the main processes that affect the interfacial adhesion between plant fibers and cement or geopolymer-based matrices; (iii) overview of fiber–matrix interfacial bonding mechanisms; (iv) pre-treatments of natural fibers used as reinforcement in cement and geopolymer composites; and (v) studies on modified natural fiber–matrix interfaces. The review finishes with concluding remarks and future perspectives arising for this technology.

## 2. Natural Fibers

**Figure 1:**
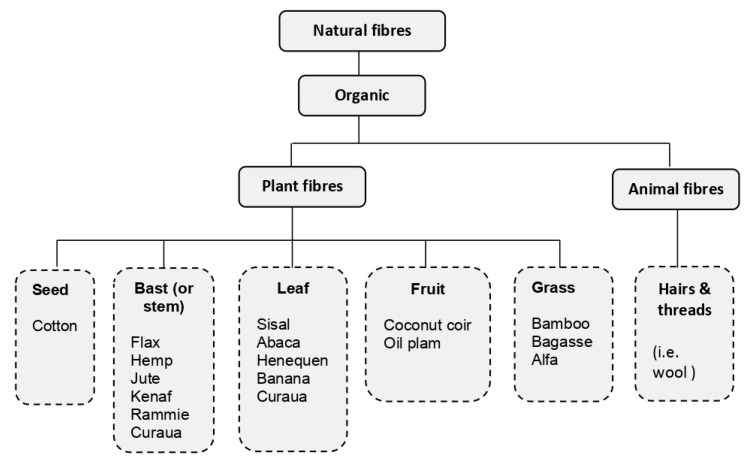
Classification of natural fibers commonly used in cement and geopolymer composite production [20,21].

Organic natural fibers can be classified in two categories: plant fibers and animal kingdom-derived fibers. Figure 1 provides a general subdivision of the organic natural fibers predominantly used as reinforcement in cement and geopolymer composites. Additionally, agricultural wastes such as rice husk ash from burning rice hulls process and bagasse from cane sugar production have also been incorporated in these composites [16,17,18,19].

### 2.1. Plant-Derived Fibers

Plant-derived fibers are themselves well-designed hierarchical composite materials basically composed of cellulose, hemicellulose, lignin, pectin, waxes, and some water-soluble materials [22,23]. The structure of a single plant fiber mainly consists of the following constituents: lumen and a central cavity, which is responsible for the water uptake behavior of the plant as well as several wall layers, which are grouped in primary and secondary walls [24]. The primary wall (P) is comprised of cellulose microfibrils with a random orientation to allow the expansion of the cells during the growth of the plant. The secondary wall (S) is subdivided into three sub-layers [24,25]. Cellulose microfibrils of the secondary wall layer present a defined orientation with a helical winding pattern [26]. Briefly, the cell walls are constituted of cellulose microfibrils coated with hemicellulose structures, which are embedded in a matrix of hemicellulose and lignin [22].

(i) Cellulose

Cellulose is essentially composed of glucose units linked in long chains. These are referred to elemental fibrils, which are joined to form microfibrils. The cellulose component is composed of crystalline and amorphous regions wherein the crystalline zone is apparently related to the core of the microfibril, while the amorphous zone is associated with the microfibril exterior [27]. The literature reports that cotton, hemp, curaua, jute, pineapple, ramie and flax fibers contain the highest percentages of cellulose (70–96%), whereas bamboo, bagasse, and coir fibers exhibit the lowest contents of cellulose (20–45%) [28,29,30].

(ii) Hemicellulose

Hemicellulose is a complex group of polysaccharides (mainly glucose, mannose, galactose, xylose and arabinose) considered as mediators between cellulose and lignin. Hemicellulose is covalently linked to lignin and bonded to the microfibril cellulose via hydrogen bonds [26,31].

Hemicellulose is generally amorphous and contains the highest proportion of accessible OH groups of the cell wall, which is associated with moisture gain capacity and lower thermal stability. It is generally agreed that hemicellulose confers viscoelastic properties to the plants, as its degradation results in the increase in stiffness and brittleness [26].

(iii) Lignin

Lignin is completely amorphous and is composed of a complex group of hydrocarbon polymers with aliphatic and aromatic components. The structure is responsible for the stiffness and the height of the plant and the protection against microbiological attack, as well as being a chemical adhesive between cell walls in the middle lamella region [32]. Lignin’s mechanical properties are lower than those of cellulose and hemicellulose. 

(iv) Pectin

Pectin is a collective name for heteropolysaccharides. Pectin confers flexibility to the plants and is predominantly found in leaves and fruits. Pectin is soluble in water in the presence of alkaline environments of ammonium hydroxide [33]. 

(v) Fats, Waxes and Lipids

Finally, fats, waxes and lipids, which consist of, among other components, different types of alcohols are insoluble in a number of solvents as well as in water at room temperature [34]. They serve as a protective barrier against microbiological attack and prevent the drying process in plants. In general, their presence negatively affects the processing, quality and wettability of natural fibers [35,36].

The microstructure and chemical composition of fibers derived from plants greatly influence their mechanical properties, wherein the content and degree of polymerization of crystalline cellulose as well as the microfibril orientation play an important role. Therefore, it can be suggested that plant fibers with higher cellulose content and a degree of polymerization as well as a lower microfibril orientation angle exhibit higher mechanical properties [33]. These findings were validated by Komuraiah et al. [37] who could demonstrate that the tensile strength was positively correlated with cellulose, but negatively influenced by lignin content. On the other hand, the specific Young’s modulus was positively affected by cellulose, hemicellulose and wax contents, while lignin and pectin contents reduced the specific fiber Young’s modulus. Regarding moisture gain, hemicellulose and lignin were shown to be largely responsible for the water uptake capacity of plant fibers while pectin had a negligible effect. A qualitative assessment of the impact of different components of the plant structure and chemical composition on the physical and mechanical performance of plant fibers is shown in detail in Table 1. 

### 2.2. Animal Fibers

Animal fibers are composed of different type of proteins. These fibers come either from mammals (hairs) or from certain invertebrates, such as the silkworm. Animal fibers are most often found from wool-bearing animals, such as sheep and goats, and fur-bearing animals, such as rabbits, mink, and fox [38,39]. These protein fibers act as protection of cells, tissues and organisms and help to facilitate elasticity, scaffolding, and stabilization. Generally, the properties of the fibers are determined by the sequence and type of amino acids forming polypeptide chains. A major part of animal fibers is represented by α-keratin, which exhibits a complex structure and different chemical compositions. Keratins are insoluble and chemically unreactive. Most mammalian fibers comprise three main morphological components: a cortex, cuticle, and medulla. The cortex builds up of main bulk part of the fiber and it determines their mechanical properties. The cuticle is the outside part of fibers and acts as a protective layer. The cuticle has a waxy coating that helps to stop penetration of water into the fiber. The medulla is the central part of the fiber. The deterioration of the medulla decreases the quality of fibers resulting in low fiber strength [40,41].

Animal fibers have high moisture absorption similar to plant fibers [42]. Additionally, it seems that the chemical composition of animal fibers facilitates the interaction with geopolymer matrices and improves the fiber resistance to alkali media. Alzer et al. [43] studied the performance of geopolymer composites reinforced with wool fibers. The findings indicated that the amino acid groups in the wool fiber reacted with the alkalis of the geopolymer matrix. Moreover, degradation reactions of wool fibers in the alkaline solution of the geopolymer resulted in the formation of lanthionine, which leads to lowering the alkali solubility of the wool to some extent. 

Although animal fibers have shown to be a promising renewable reinforcement and could display improved alkali resistant performance in comparison with plant fibers, more studies are needed to elucidate their application in cement and geopolymer composites. In fact, the number of studies reporting the use of animal fibers as reinforcement of geopolymer and cement matrices is very limited. The present review therefore mainly focuses on composites reinforced with plant-derived fibers.

## 3. Interfacial Adhesion between Plant Fibers and Cement and Geopolymer-Based Matrices

### 3.1. General Factors Affecting Interfacial Adhesion

In matrices based on Portland cement, plant fibers are generally subjected to three different actions that negatively affect their adhesion with the matrix:

(i) Debonding of fiber/matrix interface due to the water-swelling process of fibers [44,45].
(ii) Progressive alkali hydrolysis starting with the degradation of amorphous zones of the fibers (hemicellulose and lignin) and finalizing with the defibrillation of the crystalline cellulose microfibrils [46,47].
(iii) Fiber mineralization due to deposition of cement hydration products, mainly calcium hydroxide, onto the fiber surface and into the lumen [46,48].

Regarding geopolymer composites, the mechanisms of natural fiber degradation and their effect on the performance of aged geopolymer composites are still not widely studied. Only few researchers have addressed this issue. A recent study performed by Ye et al. [49] concluded that higher contents of cellulose in the fibers led to a denser structure and ductile failure of metakaolin-based composites. However, higher concentration of hemicellulose and lignin reduced not only the compressive and flexural strength, but also increased the porosity of the matrix. FTIR spectra of geopolymer reinforced with 20% of pure hemicellulose showed the presence of a new peak at around 1610 cm^−1^ associated with carboxylate anion (COO-). They correlated this peak with the poor performance of the geopolymer composite where generation of carboxyl acids from the alkaline degradation of hemicelluloses lowered the degree of geopolymerization by decreasing the alkaline environment required for the geopolymerization process. In another study, Alshaaer et al. [50] performed an aging study for 20 months at ambient conditions in metakaolin-based geopolymers reinforced with unmodified luffa fiber. They found that aging increased the flexural strength in nearly 12%, thus, stressing that the highly alkaline conditions of the metakaolin geopolymer synthesis did not degrade the luffa fibers. 

More details of the plant fiber deterioration mechanisms are described in the following sections. The discussion will be mainly focused on the degradation in cementitious matrices as there are only few studies in the geopolymer field.

### 3.2. Effect of Plant Fiber Moisture on Composites

**Figure 2:**
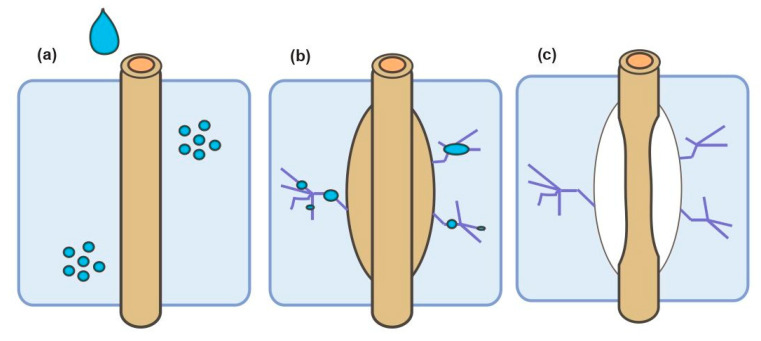
Effect of water at the plant fiber–matrix interface. (**a**) Water diffusion into the composite; (**b**) swelling of fibers after moisture adsorption leading to microcracks within the matrix; (**c**) ultimate fiber–matrix debonding. Based on reference [53].

The inherently polar-hydrophilic nature of plant fibers is one of the major limitations to their successful use in reinforcing cement and geopolymer matrices [51]. The sensitivity to moisture of plant fibers could be explained in terms of the accessibility to OH groups in the cell wall. Cellulose, hemicellulose and lignin contain different amounts of hydroxyl groups on their surface, e.g., hemicellulose (largely amorphous) possesses the highest content of OH groups available to interact with adsorbed water molecules, followed by lignin, which is completely amorphous but is characterized by a lower concentration of OH groups. In the case of cellulose, the OH groups located in the crystalline part (primarily in the core of microfibrils) are considered to be inaccessible, and they are not involved in hydrogen bonding with adsorbed water molecules. However, the OH groups present in the amorphous cellulose zone, and generally located on the surface of the microfibrils, are available to interact with adsorbed water. Thereby, OH groups contained in the amorphous region of the cell walls are responsible for moisture absorption and dimensional instability of the plant fiber [26,52]. However, some studies have outlined that the dimensional expansion of the plant fiber caused by swelling is more predominant in the cross-section of the fiber (40–60%), whereas its longitudinal expansion is about 2–3% [44]. Several investigations have been focused on the effect of moisture in cement composites reinforced with plant fibers. Degradation processes take place when the swelling and shrinking of the plant fiber induces stress at the interface region, leading to microcracking phenomena in the matrix surrounding the fiber, as detailed in Figure 2 [53]. It has been reported that water molecules absorbed onto the hydrophilic groups of the plant fiber form a large number of hydrogen bonds, which create a physical barrier between the fiber and the matrix, leading to weakening of the interface adhesion and fiber debonding [54,55,56]. Generally, the increase in fiber moisture content results in reduced modulus of elasticity and strength, accompanied by the formation of large voids in the final composites and a predominant fiber pullout failure mode [44,57,58]. 

### 3.3. Plant Fiber Alkaline Degradation

**Figure 3:**
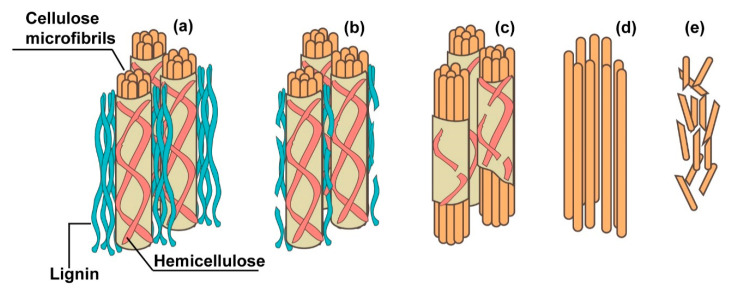
Schematic diagram showing the alkaline degradation mechanism of plant fibers. (**a**) Plant fiber compounds; (**b**) degradation of lignin and partly hemicellulose; (**c**) degradation of hemicellulose; (**d**) stripping of cellulose microfibrils; (**e**) failure of cellulose microfibrils. Based on reference [46].

The amorphous components of plant fibers suffer various degrees of degradation when exposed to an alkaline environment, as illustrated in Figure 3. Wei et al. [46] summarized in four steps the alkaline degradation of plant fibers in cement matrices: (i) degradation of lignin and partly of hemicellulose; (ii) complete degradation of the hemicellulose contributing to the loss of integrity and stability of the fiber cell wall; (iii) stripping of the cellulose microfibrils; and (iv) failure of the cellulose microfibrils, therefore leading to the complete degradation of the plant fiber. In this regard, several studies have reported that alkaline hydrolysis affects the loss of integrity of the fiber–matrix interface zone due to decomposition of the hemicellulose and amorphous region of cellulose fiber chains, thereby compromising the mechanical properties and durability of plant fiber-reinforced cement composites [48,59,60]. Furthermore, when the alkaline attack proceeds, the hydration cement products such as C-S-H and calcium hydroxide penetrate the cell wall of the fibers. It is interesting to note that Singh et al. [61] detected that the extent of the alkaline attack was more severe in a calcium hydroxide solution than in sodium hydroxide environment, even when considering that the later exhibited a higher pH. It was suggested that the presence of calcium ions leads to additional degradation due to a mineralization process. Another noteworthy investigation [62] studied the incorporation of silica fume and low-calcium fly ash as partial replacements of Portland cement to prepare composites reinforced with plant fibers. The purpose of the study was to evaluate the influence on alkalinity of the matrix due to the addition of supplementary cementitious materials. They observed a reduced alkalinity in matrices dosed with silica fume, in contrast to the limited reduction of alkalinity displayed by the incorporation of low-calcium fly ash. Additionally, Wei et al. [63] indicated that the incorporation of metakaolin prevented both mineralization and alkali hydrolysis of plant fibers in cement composites to a certain extent. 

### 3.4. Plant Fiber Mineralization

Mineralization of plant fibers is generally caused by the migration of calcium hydroxide (CH) and Ca^++^ ions from the cement matrix and pore solution to the wall cell, lumen and voids of the plant fiber, wherein CH crystallizes. Although the precipitation of these cement hydration products in the fiber vicinities increases the affinity of the fiber surface to cement matrix, the crystallization process due to mineralization corrodes the cellulose microfibrils and damages the bonding between the various fiber components [46,64]. Consequently, such composites displayed a predominant fiber rupture failure due to the embrittlement of the fiber, accompanied by a lower ductility and toughness as well as reduced flexural strength capacity [60,65]. Moreover, such changes are accompanied by partial degradation of plant fibers, resulting in the deterioration of the fiber–matrix bonding and compromising the durability properties of the composites [59].

## 4. Interfacial Bonding Mechanisms

### 4.1. Overview of Acting Mechanisms

The microstructure of composite materials includes fibers (reinforcement), the matrix and the interface. The function of the matrix is to protect the fibers from the negative impact of the environment and transfer the load to the fibers [66]. This protection is conferred by thin layers present between the fiber and the matrix forming the interface. The properties and thickness of the interface have a significant role in stress transfer and affect the mechanical properties of the reinforced composite materials. 

**Figure 4:**
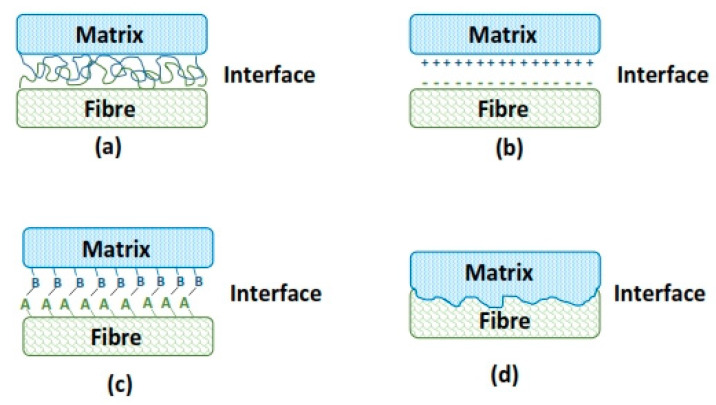
Fiber matrix interface bonding mechanisms. (**a**) Interdiffusion; (**b**) electrostatic adhesion; (**c**) chemical bonding; (**d**) mechanical interlocking. Open access under terms and conditions of the Creative Commons Attribution (CC BY), reference [69].

In general, several adhesion mechanisms can be simultaneously active depending on the specific bonding situation at the fiber–matrix interface [67,68]. The adhesive bonding theory is generally described by the following mechanisms: mechanical interlocking, electrostatic, interdiffusion, and chemical bonding, as shown schematically in Figure 4. 

### 4.2. Assessment of Bonding Mechanisms in Cement and Geopolymer Composites

Mechanical interlocking and chemical bonding are the most common and relevant phenomena displayed in cement and geopolymers composites. Some studies have shown that alkaline treatment provided a rough surface on the plant fibers treated, therefore facilitating the mechanical interlocking of the fiber with the matrix which in turn led to the increase in the mechanical properties of composites reinforced with such modified fibers [7,14,15,70]. Regarding the chemical bonding mechanism, studies on cement composites reinforced with silane-treated fibers have suggested that silane coatings enhanced the cement adhesion onto the fiber likely due to the polysiloxane network formed at the fiber surface, resulting in a large number of active functional groups that could react chemically with the matrix materials by forming stable bonds [71,72].

## 5. Pre-Treatments of Natural Fibers Used as Reinforcement in Cement and Geopolymer Composites

### 5.1. Thermal Treatments

Thermal modification can be defined as chemical and physical changes produced on natural fibers due to the application of temperature, wherein different process variables have significant effects upon fiber properties. The most important variables include the following: duration and temperature of treatment, atmosphere type, closed versus open systems, sample dimensions, and the use of catalysis [52]. Most authors [73,74,75] have reported improvements in dimensional stability, microbiological attack, and reduced hygroscopicity of thermally treated natural fibers, as well as enhanced fiber–matrix interactions. The thermal treatments performed by the cement and geopolymer industry on natural fibers might be categorized mainly as (i) cycles of wetting and drying referred to hornification; (ii) heat treatments; and (iii) hydrothermal process where fibers are heated in a liquid or steam medium. More details of these treatments are discussed in the following subsections.

#### 5.1.1. Influence of Heating Processing Parameters on Natural Fibers

As time exposure and heating temperature increase, strong chemical and physical modifications on plant cell walls are induced. In particular, it is well determined that cellulose is more thermally stable than hemicellulose and lignin [26]. In broad terms, hemicellulose, which is amorphous and of low molecular weight, could begin to decompose at temperatures as low as 100 °C [76], leading to increased crystallinity due to degradation/rearrangement of the amorphous content. In this respect, Fengel and Wegener [26] suggested a probable thermal degradation model where severe hydrolytic depolymerization in hemicelluloses takes place to form products such as acetic acid and furfural. At higher temperatures (around 160 °C), studies have reported lignin degradation accompanied by the release of various molecules (vanillin, eugenol, and guaicol, among others) [77,78]. It is generally accepted that lignin decomposes over a broad temperature range owing to the different thermal stabilities presented by their oxygen functional groups [79]. Additionally, it has been observed that crystalline cellulose degrades in the temperature range of 300–340 °C with a concomitant decrease in the degree of crystallinity [80]. With extended heating, cellulose depolymerization occurs to form alkaline-soluble oligosaccharides. Furthermore, the amorphous region of cellulose is less thermally stable and probably exhibits similar thermal behavior compared to amorphous hemicellulose. Consequently, Shafizadeh et al. [81] found reductions in the degree of polymerization of cellulose at a low temperature of 150 °C. Related to water evaporation during cellulose heating, Tang and Bacon [82] concluded that reversible physical desorption of water occurs at temperatures from 25 °C to 150 °C, while the loss of chemical water started at 220 °C according to studies of Scheis et al. [83].

On the other hand, investigations on heating processing parameters recommended to perform thermal treatments in open systems to avoid accelerated fiber degradation owing to the formation of volatile extractives and acids produced by the decomposition of the polysaccharides present in the cell wall. Furthermore, providing an inert atmosphere free of oxygen prevents oxidative processes that can negatively affect mechanical behavior [84]. In addition, Fengel and Wegener [26] also suggested the use of steam-heating to improve the heat transfer into the interior of samples with large dimensions. 

#### 5.1.2. Description of Thermal Treatments on Natural Fibers as Reinforcement of Cement and Geopolymer Composites

(i) Hornification

Hornification refers to the mechanism of irreversibly removing water from the cell wall fiber [85]. This reduction in water retention could be obtained by repeating cycles of wetting and drying. This phenomenon can be typically described as immersing the fibers in water until reaching their maximum absorption capacity followed by a drying process generally set at moderate temperatures (60–80 °C) for a certain period of time [86,87,88]. During the drying stage, a rearrangement of the polysaccharide cellulose chains takes place, in which microfibrils move closer to each other due to the water drainage resulting in the formation of irreversible or partially irreversible hydrogen bonds. Therefore, the fiber becomes more resistant to delaminating forces because most hydrogen bonds either do not reopen or react any longer even if they are damped once again. When subsequent cycles occur, the fiber cavity (lumen) could collapse and most capillary voids in the lamellar structure of the cell wall become closed. As a consequence, an intensely bonded fiber structure is obtained due to increased connection of interfibril hydrogen bonding. This process is supposed to be irreversible, that it is to say, the original water-swollen state is not regained in the presence of water [89]. However, some studies have partially shown restoration of the swelling capacity of hornified fibers by adding agents like sucrose or glycerol or performing alkaline heating [90]. 

According to some studies, the use of hornified plant fibers denoted significant benefits as reinforcement of cement composites owing to their reduced water absorption, thus diminishing the incrustations of calcium hydroxide on the surface and lumen of the fibers. This mechanism can minimize the characteristic cellulosic degradation present in cement alkaline environments [52]. Furthermore, fiber dimensional stability improves the fiber–matrix interface, therefore increasing mechanical performance and durability of the composites [86]. The aforementioned behavior was supported by results of Ferreira et al. [91,92] demonstrating in pullout tests that cement composites reinforced with sisal fibers submitted to ten cycles of wetting and drying enhanced the adhesion bond strength and frictional bond strength by about 40% and 50%, respectively, in contrast with untreated fiber composites. Similarly, Lima and co-workers [93] obtained an increase of 55% in interfacial bond strength of cement matrices reinforced with sisal fibers subjected to 10 cycles in comparison to untreated fiber composites. Additionally, hornification experiments on cotton and pulp fibers used as reinforcement in cement matrices have been reported [86,87,94]. It was found that cement composites reinforced with hornified fibers exposed to 4 cycles exhibited an increase in flexural strength of 8% (kraft pulp), 16% (cotton linters), and 19% (eucalyptus pulp) in contrast to composites reinforced with raw (untreated) fibers.

Durability studies on cement composites reinforced with hornified fibers have also been carried out by artificially aging composites through wet/dry cycles. Claramunt et al. [86] stressed that the hornification process enhanced the mechanical performance of aged composites, in which hornified (four cycles of wetting and drying) kraft pulp and cotton cement composites displayed 13% (kraft pulp) and 21% (cotton) higher values of flexural strength, and 20% (kraft pulp) and 10% (cotton) higher values of compressive strength in comparison with aged untreated composites. In contrast, recent research [95] has revealed that the success of the hornification process depends on the fiber type. It was found that cement composites reinforced with hornified curaua and jute fibers submitted to 10 cycles of wetting and drying exhibited a reduction in the fiber–matrix bond by approximately 33% and 51%, respectively, compared to untreated composites, whereas composites reinforced with hornified sisal fiber (10 cycles) displayed an increase in interfacial bond of 40%. The authors suggested that the decrease in the fiber–matrix bond could be correlated to a surface inactivation process, wherein lignin might block the formation of hydrogen bonds, therefore preventing interfiber crosslinking. Furthermore, SEM analyses showed a severe damage on jute fiber, indicating that a critical number of cycles of wetting and drying should be considered.

Some modifications have been made on the hornification process in order to enhance its effectiveness. For example, Claramunt et al. [96] evaluated different wet and dry methods on nonwoven fibers as reinforcement in cement composites. They compared wetting in water at room temperature versus steam at 120 °C in an autoclave and drying at 60 °C versus heating at 160 °C plus ironing (at 190 °C for 2 min). The best combination was found to be wetting in water at 20 °C followed by a drying stage of heating at 160 °C plus ironing, repeating the cycles five times. An improvement in bonding force of nearly 44% was observed on optimized hornified nonwoven fiber-reinforced cement composites compared with untreated nonwoven composite. On the other hand, Ferreira and co-workers [97] evaluated the feasibility of applying hornification of sisal fibers (10 cycles of wetting and drying) prior to chemical impregnation with styrene butadiene polymer. The group detected a synergistic effect, in which cement composites reinforced with hybrid-treated (hornification + polymer coating) sisal fibers showed the highest values of interfacial bond strength (0.86 ± 0.14 MPa) compared with hornified sisal composite (0.42 ± 0.08 MPa), polymer coated sisal composite (0.49 ± 0.12 MPa), and untreated sisal composite (0.30 ± 0.08 MPa).

(ii) Heat Treatment

Several studies have been performed on analyzing the effects that heat treatment exerts on natural fibers and their cement composites. Wei et al. [98] found that the tensile strength and Young’s modulus of sisal fibers treated at 150 °C for 8 h in a ventilated oven were improved by 45% and 70%, respectively, in contrast to untreated fibers. The enhanced mechanical properties of sisal fiber were attributed to the increased crystallinity of the cellulose and correlated to the notable durability of their concrete composites subjected to 30 wetting/drying cycles, which displayed a tensile and compression strength reduction of 21% and 17%, respectively, in comparison with untreated sisal composites, wherein the loss in mechanical behavior was higher with values of 34% for the tensile strength and 25% for the compression strength.

Another variant of heat treatment is related to pyrolysis. This is a common method used to decompose organic materials in an inert atmosphere (without oxygen) by preventing combustion [99]. In another study [100] the authors examined the application of pyrolysis at 200 °C for 2 h on banana and sugar cane bagasse fibers with the purpose of rearranging carbohydrates present in cellulose and hemicellulose as well as to achieve dehydration of the fibers. It was observed that the tensile strength on bagasse and banana fibers was augmented by a factor of 3 and 5, respectively, after pyrolysis treatment. In contradiction with the superior mechanical performance exhibited by pyrolyzed fibers, the mechanical properties of their cement composites did not show significant improvement.

(iii) Hydrothermal Treatment

This method refers to the lignocellulosic changes caused by heating fibers in an aqueous medium such as water or under steam flow. Depending on the operational conditions, the depolymerization of the hemicellulose—as well as that of cellulose and lignin by similar hydrolysis reactions—is expected to yield sugar oligomers and degradation products [101]. Hydrothermal modification by heating natural fibers in water, followed by washing and drying processes is a pre-treatment used by the cement industry with the purpose of removing water-soluble sugars and extractives (tannins, resins, phenols, etc.) from the plant fibers, which act as retarding agents during the setting of the cement paste, consequently diminishing the mechanical behavior of concrete composites [102]. Research reported in reference [103] proved that boiling coconut coir fibers in water for 2 h followed by a washing procedure was an effective method to remove soluble chemicals, and hence to improve the flexural strength (by 283%) in contrast to cement boards reinforced with raw coconut fibers. Likewise, investigations [104] on the feasibility of using diss fiber (a vegetable species from the Mediterranean region) determined that boiling the fibers in water for 4 h prior to a washing step was a successful procedure to eliminate water-soluble components, in particular sugar, which decreased from 31% for untreated diss fiber to 1% for boiled-and-washed diss fiber. The findings could explain the greater flexural and compressive properties displayed by the cement composites reinforced with treated diss fibers. 

### 5.2. Biological Treatments

Substantial research has been undertaken to find environmentally friendly methods for fiber modification. In this aspect, biological treatments of plant fibers are gaining popularity owing to low-energy processing, milder reaction conditions, the possibility to implement recycling systems, and improved properties of fibers achieved [105,106]. These technologies involve the use of enzymes, fungi, bacteria or other biological sources obtained from animals, plants or microbes to selectively remove non-cellulosic components (e.g., pectin, hemicellulose and lignin) from the plant fiber [24,107]. 

#### 5.2.1. Enzymes

Enzymes are biological catalysts that accelerate biochemical reactions. The main role of the enzymes on natural fiber modification is to separate the fiber from its non-fiber components while improving the cleanliness, homogeneity, surface area and water resistance [108]. The most common enzymes applied on fibers in the composites industry are cellulases, pectinases, laccases, and xylanases [109]. Cellulases are hydrolytic enzymes that catalyze the breakdown of cellulose to smaller oligosaccharides and finally glucose. Their activity should be limited to preserve the fiber strength, and, consequently, their use is indicated to a degrade amorphous cellulose [110] before deteriorating crystalline cellulose [111]. Cellulases are generally employed to process cotton and other cellulose-based fibers [106]. Pectinases are a complex group of enzymes involved in the degradation of pectin compounds, resulting in the separation of fibers and non-fiber components [112]. Laccases are responsible for the degradation of lignin [113], while xylanase enzymes break down the hemicellulosic component around the fiber [114].

#### 5.2.2. Fungi

In the biocomposites field, white rot fungi from the Basidiomycetes species have been the only organisms able to degrade lignin efficiently [115,116]. They synthesize extracellular oxidases that degrade not only lignin but also a broad range of non-cellulosic materials including resin acids, fatty acids, and oils resulting in enhanced fiber strength [117,118,119]. 

#### 5.2.3. Bacteria

This method involves the culturing of specific bacteria, mainly Acetobacter species such as *A. xylinum*, in the presence of the plant fibers to deposit pure nanosized cellulosic materials on their surface. Coating the fibers with bacterial nanocellulose results in enhanced fiber–matrix adhesion through mechanical interlocking [120,121,122]. 

An increasing number of studies has been carried out on evaluating biological treatments on natural fibers for the polymer industry demonstrating great potential as reinforcement agents. For instance, George et al. [108,114] investigated the performance of hemp and flax fibers treated with the following enzymes: hemicellulose (xylanase), pectin (PG: polygalacturonase), lignin (laccase), and cellulose (xylanase + cellulase) at enzyme-specific conditions, which has been explained in detail in reference [108]. It was found that xylanase and polygalacturonase were effective in removing hemicellulose and pectin materials, generating better thermal properties and water resistance capacity. Although the enzymatic method produced nearly 25% of degradation in the natural fibers, it did not compromise the composite performance. On the other hand, Pickering et al. [123] tested the activity of different Basidiomycetes (white rot fungi), Zygomycetes and Ascomycetes fungi species on hemp fibers. They outlined that although fungi treatment reduced the tensile strength and Young’s modulus in all hemp fibers, the resulting polypropylene composites exhibited higher tensile strength compared with untreated fiber composites due to improved wettability and mechanical interlocking with the polypropylene matrix. These results confirm the need to investigate both the effect of biological treatments on the natural fibers itself and on the resulting composites. Hence, there is still considerable research to be carried out on how enzymatic and fungi treatments on natural fibers influence the properties of cement and geopolymer-based composites. 

In the context of the coating of plant fibers with bacterial cellulose, Mohammadkazemi et al. [124] have reported advances in studying the performance of cement composites reinforced with bagasse fiber coated with cellulose harvested from bacteria *Gluconacetobacter xylinus*. The findings showed that composites reinforced with coated cellulose fiber exhibited a 68% increase in flexural strength, a 40% increase in internal bonding strength, and nearly 70% increase in fracture toughness as compared with untreated fiber cement-based composites. Their superior behavior was attributed to extra accessible hydroxyl groups of bacteria cellulose, which provided a strong interface with the cement matrix. Furthermore, the high crystallinity of bacterial cellulose prevented fiber mineralization, a characteristic mechanism of deterioration presented by cellulose-reinforced cement composites. Extensive literature related to bio-treatments on natural fibers can be found in previous publications [24,105,106,118,120,121].

### 5.3. Chemical Treatments

These types of treatments remove impurities from the natural fiber surface, thus improving fiber–matrix adhesion. According to some studies, chemical treatment could stimulate the active hydroxyl groups of natural fibers to react with the matrix [101,102]. Many researchers have used different chemicals to modify the fiber surface, such as alkali, silane, isocyanate and formaldehyde agents to reinforce geopolymer and cement matrices [125,126,127,128,129]. Alkali treatment is one of the oldest and best-known methods for the modification of natural fibers [130]. The most reasonable alkali solution for fiber treatment consists of sodium hydroxide (NaOH) aqueous solution. The vital modification performed by alkali treatment is the removal of the fiber constituents including hemicellulose, lignin, pectin, fat, and wax, which expose cellulose and increase surface roughness. Moreover, appropriate alkali treatments modify the cellulose structure and increase cellulose crystallinity, thereby improving interfacial bonds between the fiber and the matrix. On the contrary, alkali treatment at higher concentration creates an excess of delignification, leading to weaker or damaged fibers [131,132,133]. The following scheme represents the established chemical reaction of NaOH with hydroxyl groups in natural fibers:

Natural fiber − OH + NaOH → Natural fiber − O^−^ + Na^+^+ H_2_O

Many studies have been performed on analyzing the effects that alkali treatment exerts on natural fibers and their geopolymer and cement composites.

Malenab et al. [15] treated abaca fiber using 6 wt.% NaOH solution for 48 h and Al_2_(SO_4_)_3_ 10 wt.% solution for 12 h. They observed that the alumina salt treatment was effective to form AlOH_3_ deposits on the abaca fiber surface. Then, they synthesized abaca fiber-reinforced geopolymer composite. The flexural strength of the alumina-treated fiber composite was improved by 65% in comparison to untreated fiber-reinforced composite. Similarly, Janne et al. [134] treated abaca fiber with 6 wt.% NaOH solution to reinforce foamed geopolymer composites. These researchers observed that the treated abaca fiber appeared rougher and more uniform on SEM images. In theory, the rough surface should facilitate the fiber–matrix interlocking. The compressive and flexural strengths of the composites containing treated fibers were found to improve from 19.6 to 36.8 MPa, and from 2.4 to 6.3 MPa, respectively. Likewise, Chen et al. [70] immersed sweet sorghum fiber into 2 M NaOH solution for 12 h prior to incorporation in fly ash-based geopolymer composites. The authors selected NaOH solution as pre-treatment of sweet sorghum fiber due to its compatibility with the alkali environment of the geopolymer matrix. The researchers explained that this treatment enhanced fibrillation and promoted surface roughness. Moreover, the NaOH treatment improved the split tensile strength and fracture toughness of geopolymer composites, whereby the split tensile test clearly showed the transition of failure mode from brittle to ductile failure. In another study, Kriven et al. [135] treated jute weave and fique fibers with 0.5 wt.% NaOH solution for 24 h and with 5 wt.% NaOH solution for 4 h, respectively, to reinforce metakaolin-based geopolymer matrices. They found that treated jute weave-reinforced geopolymers exhibited higher tensile strength and elongation at breakage when compared with samples reinforced with untreated fibers. The tensile strength of the composite was improved from 8.8 to 14.5 MPa. They also noticed that the alkali solution removed the hemicellulose part from the fiber, and the fiber surface became rough, resulting in increased friction between the fiber and the geopolymer matrix. On the contrary, Ribeiro et al. [7] investigated metakaolin-based geopolymer composites reinforced with short micro-bamboo fibers. The bamboo fibers were treated with deionized water and NaOH solutions. The authors reported that the flexural strength in both alkaline- and water treated fiber composites achieved similar values (around 8 MPa). On the other hand, Zhou et al. [136] evaluated the behavior of cement composites reinforced with hemp fiber. The hemp fibers were treated with a 2 wt.% Ca(OH)_2_ alkaline solution for 14 h. The authors reported that treated hemp fiber-reinforced composites showed higher compressive strength, tensile strength and fracture toughness by 10%, 17%, and 7–13%, respectively, in contrast to unmodified fiber composite. Additionally, the modified fiber composites exhibited 11% less brittle and 10.8% more ductile behavior compared with unmodified fiber composites. Correspondingly, Sawsen et al. [137] used flax fiber to reinforce cement matrices, wherein flax fibers were immersed in 6 wt.% NaOH solution for 48 h. The modified flax fiber-reinforced composite displayed a flexural strength 27% higher (at 90 days) than that achieved by an unmodified fiber cement composite.

#### 5.3.1. Silane Treatment

Silane coupling agents commonly improve the degree of cross-linking between the fiber and the matrix in composites [133,138]. A silane coupling agent is an organic compound with a chemical formula SiH_4_. Silane coupling agents are molecules with two functional groups, wherein the first functional group should react with hydroxyl groups of cellulose, and the second functional group should react with the matrix. Moreover, silane coupling agents have been an effective modifying method for natural fiber–matrix interfaces by reducing the hydroxyl groups of the fiber [139,140,141,142]. This treatment has been shown to contribute to the improvement of mechanical and water resistance of cement composites as well as enhancing the interfacial adhesion between the fibers and the matrix [71,143]. Bilba et al. [71] modified bagasse fibers with alkyltrialkoxysilane RSi(ORO)_3_ (S1) or dialkldialkoxysilane R_2_Si(OROO)_2_ (S2). The silane solutions used varied from 0.5% to 8% by volume in order to enhance their effectiveness. The researchers observed that the bagasse fibers treated by 6% volume of silane solution enhanced the fiber dimensions and porosity, therefore decreasing the water absorption and setting time of cement composites.

#### 5.3.2. Formaldehyde Treatment

Literature reports [43] have shown that wool fibers degrade in high-alkali environments. In order to reduce wool alkaline deterioration, researchers have performed formaldehyde treatments. Formaldehyde is a cross-linking agent wherein its molecules react with wool fiber to form numerous alkali-resistant cross-linkages, therefore reducing the alkaline wool solubility by 66% compared with untreated wool. Furthermore, studies have shown that formaldehyde-treated wool fiber improved dimensional stability and recovery capacity, as well as removing lipids and fatty acids from the surface of the wool fiber [111,131]. Formaldehyde treatment of wool fiber was investigated by M. Alzeer et al. [43]. It was shown that formaldehyde treatment improved the alkali resistance, thermal stability and tensile strength of the geopolymer composites reinforced with chemical treated wool. Moreover, this treatment prevented the formation of free polysulfide ions.

#### 5.3.3. Isocyanate Treatment

Polymethylene-polyphenyl isocyanate (PMPPIC) is a compound containing the isocyanate functional group –N=C=O, which is greatly susceptible to react with the hydroxyl groups of cellulose and lignin. This treatment is effective and could be used to modify both fibers and matrix. The reaction of the isocyanate chemicals depends upon the catalysts and the temperature. The core drawback of this method is the toxicity of the chemicals used. Isocyanate works as a coupling agent and has been used in fiber-reinforced composites [144,145,146]. In another study [128], the authors investigated the modification of cellulose pulp fiber by using aliphatic isocyanate (n-octadecyl isocyanate (Al)) to reinforce a cement matrix. The treatment improved the modulus of elasticity in the treated composites after 400 aging cycles: from 19.4 to 22.6 GPa. It was also observed that a reduction in water absorption capacity and apparent porosity of fiber-reinforced composites occurred. Similarly, Tonoli et al. [147] treated eucalyptus pulp fibers by using methacryloxypropyltrimethoxysilane (MPTS), aminopropyl tri-ethoxy silane (APTS) and n-octadecyl isocyanate). The researchers noticed that the treatment showed significant influence on the fiber–cement interface. MPTS-treated fiber decreased the water absorption of the fiber cement composite and improved the dimensional stability in contrast to the APTS-modified fiber-reinforced composite.

### 5.4. Assessment of Pre-Treatments Methods on Interfacial Properties

In this section, different pre-treatment methods for natural fibers have been discussed, which have been developed to improve the interfacial properties of composites, wherein cost-effective and environment-friendly parameters could influence their wider applicability. Moreover, it seems that the type of natural fiber greatly affects the efficacy of the selected treatment due to the variability of composition among the different plant species. In this respect, for instance, hornified cotton and sisal fibers have displayed enhanced performance in cement composites in contrast to those composites reinforced with hornified jute fibers, whereby the quantity of lignin in each type of fiber might influence the formation of hydrogen bonds, and consequently the interfiber crosslinking and the success of the hornification treatment [86]. On the other hand, geopolymers reinforced with alkaline treated jute fibers exhibited enhanced mechanical properties. In general terms, chemical treatments could play a major role in the interfacial properties of the fiber-reinforced composites wherein coupling agents as silanes have been found to increase the interfacial properties due to the cross-links between silanes, treated fibers and matrix that improve the tensile and flexural strength of the final composite [142,148]. On the other hand, thermal treatment on plant fibers affects their physical, chemical and mechanical properties, including water content, chemistry, and cellulose crystallinity, resulting in enhanced mechanical properties of the modified fiber [149,150]. However, these improvements sometimes result in more modest increases in the composite properties. For instance, Cao et al. [151] reported an increase of over 60% in the tensile strength of kenaf fiber due to increased fiber crystallinity, in contrast to the 10% of improvement in tensile strength of the final composite. Additionally, biological treatments aim at providing a clean surface and reducing the hydrophilic properties inherent in plant fibers to improve the compatibility between lignocellulosic fibers and hydrophobic composite matrixes. In this aspect, a comparative study on plant fiber treated with chemical, physical and biological methods was performed by Jayamani et al. [152], wherein the fungal treatment was the most effective method to improve the fibers’ characteristics in comparison to heat treatment. Another comparative study on the influence of thermal and chemical pre-treatments on plant fibers was performed by Ferreira et al. [97]. The highest improvement was achieved by cement composites reinforced with polymer-coated fibers with an increase of 63% in the interfacial shear strength, whereas the composite reinforced with hornified fibers displayed an improvement of 40% in comparison to composites reinforced with raw fibers. Research has been also conducted on the application of a combination of hornification and polymer impregnation pre-treatments on plant fibers, wherein the interfacial shear strength exhibited an increase of 183% with respect to the unmodified composite. The greater adhesion bond was explained by the dimensional stability promoted by hornification and the chemical interaction between the polymer coating and the matrix.

The assessment of the literature thus indicates that more comparative studies on the behavior of different types of fibers in composite materials under specific treatments should be conducted in order to clearly elucidate the benefits and drawbacks that result from each pre-treatment method and thus to ascertain their suitability for applications, leading to cement or geopolymer matrix composites with superior properties.

## 6. Studies on Modified Natural Fiber-Matrix Interfaces in Cement and Geopolymer Composites

Over the years, different approaches have been presented and developed to evaluate the properties of the fiber-matrix interface in composite materials [153]. In this section, several commonly applied techniques, which serve to gain knowledge on the physical and chemical properties of modified fibers and on the mechanical behavior of composites based on the properties of the interfaces, are briefly introduced. 

### 6.1. Microstructural Studies

Scanning electron microscopy (SEM) provides qualitative analysis related to interfacial bonding in composites. In this aspect, the SEM technique is generally used to characterize the surface morphology of natural fibers; fracture mechanisms, such as fiber pullout, debonding, and fiber fracture at failure surfaces; and the microstructure at fiber–matrix interfaces [153]. 

Depending on microstructural alterations of the fiber subjected to pre-treatments, the adhesion with the matrix could be reduced. Observations are mainly based on detecting smooth or rough surfaces, fiber degradation, and deposition of materials from the matrix onto the fiber. It is commonly agreed that a rough surface rises the number of anchorage points, therefore providing better mechanical interlocking with the matrix [153]. Related to natural fiber deterioration, several investigations on cement composites have reported natural fiber degradation through alkaline attack, particularly in cement matrices rich in calcium ions [46]. Additionally, in cement composites, the deposition of calcium hydroxide on the surface of natural fibers and into their lumen cavity, resulting in fiber mineralization, embrittlement, and loss of composite ductility, has also been reported [13].

Regarding the interfacial zone, the microstructure of the matrix surrounding the fiber is another significant point of interest. When a dense microstructure is developed at the interfacial zone, a fiber fracture mechanism could occur, while a “more open” microstructure could induce failure by fiber pullout. Additionally, interfacial gaps around the fibers are considered a dimensional instability leading to lower bond with the matrix [154].

Qualitative evidence has been collected by several studies regarding the effects that thermal, chemical and biological treatments of natural fibers exert on the fiber–cementitious matrix interface. On the subject of thermal treatments, it has been observed that hornification increased packing accompanied by a reduction in the fiber lumen cavity, consequently decreasing the fiber water absorption capacity and enhancing dimensional stability [91,94]. Moreover, Ballesteros et al. [87] found that hornification prevented the penetration of cement hydration products in the lumen of pulp treated fibers. In contrast, Ferreira et al. [95] detected that hornification treatment on curaua and jute fibers caused delamination and decreased the fiber–matrix bond. 

As far as heat treatment on natural fibers is concerned, Wei et al. [98] indicated that heating sisal fiber to 150 °C for 8 h in a ventilated oven barred the mineralization process, wherein no signs of calcium hydroxide deposits in the fiber lumen or voids were found. They concluded from XRD analysis that heat treatment would lead to higher crystallinity, thus preventing a petrification process. On the other hand, heat treatments could result in increased surface roughness of fibers, which was attributed to the removal of impurities, leading to better mechanical interlocking [80,84,155].

Turning to chemical treatments, it has been also indicated that chemical agents such as silane groups and NaOH solutions tend to roughen the plant fiber surface. The former treatment was shown to cause striations on the fiber surface upon drying and cracking process of the wall microfibrils [71], and the later treatment acted by removing non-cellulosic polysaccharide [137,156]. In addition, densification mechanisms at the fiber–cementitious matrix interface could take place when chemical agents react with natural fiber groups and also with cement hydration products. For example, according to [157], treating coir fibers with a wetting agent (2-ethylhexanol) might effectively improve the fiber–matrix bonding strength due to reaction of the chemically modified coir fiber with the cement products by forming a ticker cementitious layer on the treated fiber [158]. Additionally, Ferreira et al. [97] observed a notable increase in roughness in alkaline treated sisal fiber contributing to a higher frictional pullout, which caused defibrillation in the fiber. A hybrid pre-treatment (hornification + styrene butadiene polymer coating) on the sisal fiber was also performed, observing some fibrillation and peeling off effects on the fiber surface during the pullout test. These findings corroborated the higher performance of modified fiber-reinforced cement composites with respect to unmodified composites. The group attributed the enhanced interfacial bonding to a synergistic combination between the chemical anchoring provided by polymer coating and the tighter packing of the fiber structure provided by the hornification process [97]. Nevertheless, coupling agents should be carefully selected on the basis of fiber and matrix chemistry, as some studies have underlined lower composite performance in the case of some chemical treatments. For instance, Tonoli et al. [13] indicated that eucalyptus pulp fiber treated with the silane agent (aminopropyltri-ethoxysilane) presented accelerated mineralization and higher embrittlement upon aging cycles in cement-based composites. Concerning biological treatments on natural fibers with bacteria strains, Kazemi et al. [124] observed that cellulose deposited on bagasse pulp fiber surfaces increased its roughness and prevented the penetration of cement hydration products into the lumen, leading to a strong interface and enhanced durability.

Contradictory results have been reported associated with the effects that alkaline treatment on natural fibers exerts on geopolymer composites. On one side, Janne et al. [134] revealed that fly ash-based geopolymer reinforced with alkaline-treated abaca fiber displayed an improved fiber–matrix interface, while Sankar et al. [8,14] detected gaps in the interface of metakaolin-based geopolymer reinforced with alkali-treated fique and jute fibers. They also indicated a predominant pullout fiber mechanism, and the propagation of cracks through the matrix deflected around the treated fibers, thereby corroborating the existence of a weak interface. Correspondingly, Ribeiro et al. [7] conducted a comparative study between metakaolin-based geopolymers reinforced with washed bamboo fiber and alkali-treated bamboo fiber. SEM micrographs revealed imprints of the pulled out washed bamboo fibers on the fracture surface of the composite, thereby confirming its higher mechanical behavior in comparison with the geopolymer composite reinforced with alkali-treated bamboo fiber. 

**Figure 5:**
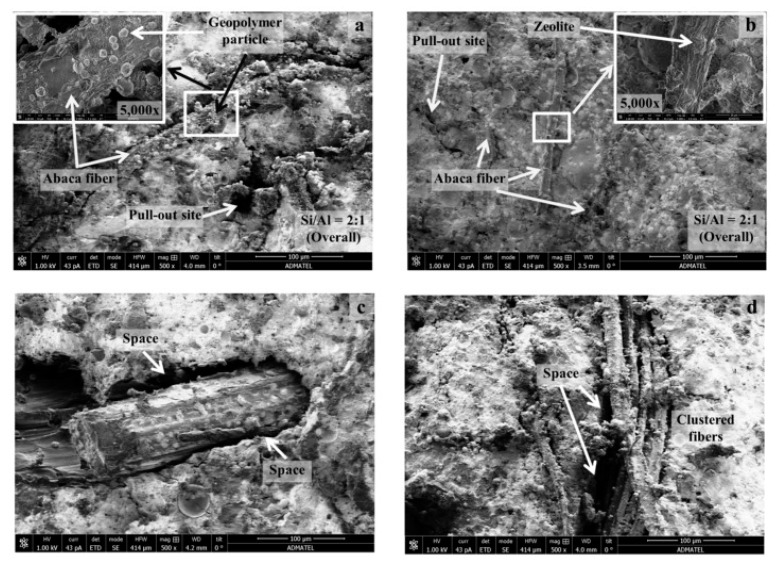
SEM images of geopolymer composite fracture surfaces reinforced with unmodified and Al_2_(SO_4_)_3_-modified abaca fibers. (**a**) Geopolymer particles observed on unmodified abaca fiber and (**c**) visible gaps at the interface between unmodified abaca fiber and the geopolymer matrix. (**b**) Geopolymer reinforced with Al_2_(SO_4_)_3_-modified abaca and (**d**) narrowed gaps detected at the interface between modified abaca fiber and geopolymer matrix. Open access under terms and conditions of the Creative Commons Attribution (CC BY), reference [15].

In contrast, chemical treatments such as impregnation of wool fibers with chemical agents such as formaldehyde led to good fiber interface performance in metakaolin-based geopolymer composites [43]. This was supported by the fiber imprint of the pulled out treated fibers. Moreover, Malenab et al. [15] found that an Al_2_(SO_4_)_3_ coating treatment on waste abaca enhanced the interfacial bond with the fly ash-based geopolymer matrix, as shown in Figure 5. It was suggested that the Al(OH)_3_ surface deposits observed on waste abaca fiber may provide extra anchoring points with the matrix. It was also shown that the interfacial gap previously observed in the untreated reinforced composites was improved after Al_2_(SO_4_)_3_ coating treatment on waste abaca fiber [15].

### 6.2. Crystallographic Studies

XRD analysis determines the crystallinity of phases present in natural fibers and is thus commonly used to indicate changes in fiber crystallinity. The most common method to calculate the crystallinity index (CrI) is detailed in Equation (1). This method uses the intensity values related to the diffraction of the crystalline structure and amorphous fraction [159]:

(1) $$\mathrm{CrI}\;\left(\%\right)=\frac{\mathrm{Icr}-\mathrm{Iam}}{\mathrm{Icr}}\times 10$$

where Icr is the intensity (peak height) of the crystalline peak at the maximum value (2θ between 22° and 23°), and Iam is the intensity at the minimum value (2θ between 18° and 19°) on the amorphous band. Malenab et al. [15] reported an increased crystallinity index (CrI) of nearly 30% in alkaline (NaOH)-treated abaca fiber compared with untreated fiber. They indicated that alkaline treatment was able to remove amorphous components, including hemicellulose and lignin, consequently allowing the arrangement of the cellulosic part, thus leading to increased crystallinity. Similarly, Ferreira et al. [97] performed several treatments on sisal fiber such as hornification, alkali treatment, polymer impregnation, and hybrid treatment (hornification + polymer impregnation) to reinforce cement matrix. They observed that the crystallinity indexes were 80% for untreated sisal, 87.8% for hornification, 88.2% for alkaline treatment, 84.8% for polymer impregnation, and 80.6% for hybrid treatment. The findings showed that the amount of cellulose content was increased in treated sisal fiber compared to untreated sisal fiber. They also detected that when sisal fiber was subjected to hornification and alkaline treatment, the crystalline fraction increased due to the partial removal of lignin and other amorphous components.

In reference [98], sisal fibers were treated by using Na_2_CO_3_ saturated solution and heat treatment at 150 °C for 4 h, 8 h and 16 h to reinforce a cement matrix. The authors indicated that the crystallinity index of untreated fiber was 20.3%, while it was 22.7% for Na_2_CO_3_ treated fiber, 22.7% for heat-treated fiber for 4 h, 26.4% for heat-treated fiber for 8 h, and 23.9% for heat-treated fiber for 16 h. The results showed that the amount of cellulose content in treated fibers increased as a consequence of the decomposition of waxes and degradation of hemicellulose compounds.

### 6.3. Fourier Transform Infrared Spectroscopy (FTIR)

In the field of composites reinforced with natural fibers, Fourier transform infrared spectroscopy (FTIR) is a versatile tool often used to monitor the following parameters: the cell wall composition of raw natural fibers, chemical surface modifications on treated natural fibers, and interactions between fiber and matrix components [153]. In general terms, FTIR spectra of plant fibers could be mainly divided in two characteristic regions: (i) the 3500 to 2800 cm^−1^ region—the vibrational modes in this range are commonly associated with the O-H and C-H stretching modes [160,161], (ii) the 2000 to 800 cm^−1^ region—in this zone cellulose, hemicellulose and lignin compounds display several peaks wherein the majority are linked to methoxyl groups (-OCH_3_, C-O-C, and C=C aromatic ring groups) present in lignin [78]. 

**Figure 6:**
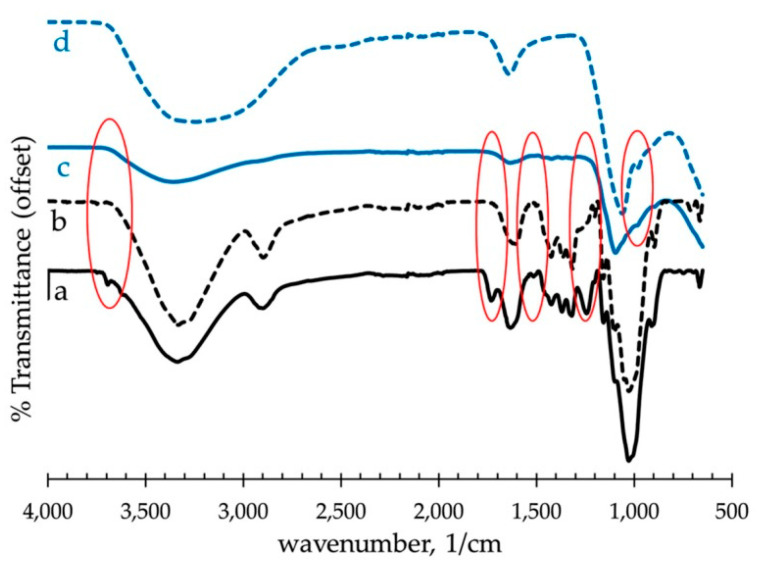
FTIR spectra of raw (untreated), NaOH-treated and Al_2_(SO_4_)_3_-treated abaca fibers: (a) untreated abaca; (b) NaOH-treated abaca; (c) NaOH + Al_2_(SO_4_)_3_ treated abaca; and (d) precipitate or residue from the spent solution of Al_2_(SO_4_)_3_ treatment. The circles indicate the loss of functional groups of raw abaca fiber due to the sodium hydroxide and aluminum sulfate chemical pre-treatments. Open access under terms and conditions of the Creative Commons Attribution (CC BY), reference [15].

Characteristic modifications in absorption bands can be identified according to the treatment performed. Related to alkaline treatment on natural fibers, the most common chemical agent used is NaOH solution. The main effects of FTIR spectra displayed by alkaline-treated plant fibers are associated with the broadening or disappearance of peaks linked to hemicellulose and lignin compounds. For instance, Malenab et al. [15] performed NaOH alkaline treatment on waste abaca to reinforce fly ash-based geopolymers, and the FTIR spectra are shown in Figure 6a–d. They detected the disappearance of the bands at 1510, 1430, and 1250 cm^−1^ attributed to the loss of phenolic groups from lignin and pectin compounds. The absence of the peak at 1430 cm^−1^ assigned to the C=O stretching vibration was also observed, which is the characteristic band of hemicellulose, as illustrated in Figure 6b. Furthermore, two additional pre-treatments on waste abaca were applied involving the deposition of aluminum sulfate (-Al_2_(SO_4_)_3_) on waste abaca and the combination of NaOH alkaline treatment followed by aluminum sulfate impregnation. It is apparent from FTIR spectra shown in Figure 6c,d the appearance of a new peak at 990 cm^−1^ associated with Al-O bonds, thus indicating the formation of Al(OH)_3_ on abaca fiber surface during aluminum sulfate treatment. Correspondingly, Ferreira et al. [97] performed an alkaline treatment on sisal fibers to reinforce cement composites. They observed the absence of the bands at 1730 and 1245 cm^−1^, associated with the stretching vibrations of C=O and C-O groups, which indicated the removal of hemicellulose and lignin constituents. The disappearance of the peak at 1630 cm^−1^ related to hydroxyl groups belonging to hemicellulose and lignin was also reported. 

With regard to thermal treatments such as hornification, Ferreira et al. [97] also observed a significant intensification of the peak around 3400 cm^−1^ attributed to the increased number of –OH bonds generated during wetting/drying. The higher intensity of the band around 3400 cm^−1^ has also been detected by other researchers in FTIR spectra [94,162]. 

Treating natural fibers with polymer agents to reinforce cement composites is gaining greater popularity. Within this context, Ferreira et al. [97] confirmed the interaction between the polymer coating agent (styrene butadiene) and the sisal fiber due to the presence of a new peak located at 1739 cm^−1^ in the FTIR spectrum, which was correlated to the C=O stretching of the ester linkage. In another study, Peruch et al. [163] examined the FTIR spectrum of sisal fibers treated with cellulose acetate polymer. They observed a reduction of the peaks located in the 1500–1300 cm^−1^ region, confirming the alteration of the fiber surface. Additionally, it was noticed that the peak at 1239 cm^−1^, associated with the C-O stretching vibration of the acyl group linked to lignin, was shifted after the polymer treatment. 

### 6.4. X-Ray Photoelectron Spectroscopy

X-ray photoelectron spectroscopy (XPS) has been used as a powerful tool to analyze fiber surfaces. XPS can be applied to a broad range of materials and provides valuable qualitative and chemical state information from the surface of the materials. Elemental identity, chemical state, and quantity of the detecting element could be determined from the binding energy and intensity of the photoelectron peak. The elementary surface composition can be determined by applying Equation (2):

(2) $$\frac{I1/S1}{I2/S2}$$

where I is the intensity of the signal, and S is associated with the atomic sensitivity factor. The subindexes (1 and 2) can correspond to carbon, oxygen, silicon or nitrogen components [128,164,165]. Henrique et al. [128] used the X-ray photoelectron spectroscopy technique to compare the composition of isocyanate-treated cellulose pulp fiber with the untreated fiber. They observed from XPS spectra that both unmodified cellulose pulp fiber and modified fiber displayed common peaks attributed to the oxygen atom (532 eV) and the carbon atom (284 eV). After treatment, they detected a new nitrogen signal at approximately 400 eV. The oxygen/carbon ratio (O/C) was changed from 0.8 to 0.63. The decrease in the O/C ratio between the unmodified and modified fibers was due to the delignification and removal of hemicelluloses from the fiber after treatment. Additionally, they noticed that the nitrogen/carbon ratio increased from 0.01 to 0.03 for unmodified and modified fibers, respectively.

### 6.5. Micro-Mechanical Fiber Adhesion Testing

The interfacial shear strength is commonly used to characterize the adhesion of a specific fiber–matrix interface system. Different experimental techniques are being applied to assess the fiber–matrix interface strength by characterizing the interfacial shear strength either indirectly or directly. Such experimental techniques developed to measure the interfacial bond include the fiber fragmentation test, the fiber pullout test, and the fiber micro-indentation test [166].

#### 6.5.1. Fiber Pullout Test

The single fiber pullout test is a well-accepted method for evaluating the interfacial strength and bond quality at fiber–matrix interfaces. Different authors have reported that increasing the interface bond between the fiber and the matrix leads to the improvement of compressive strength and toughness. However, beyond a certain limit of bond strength, usually toughness begins to decrease due to fiber rupture. On the other hand, the fiber pullout failure mechanism, achieved by optimized interfacial bond strength, is associated with higher composite toughness.

The fiber pullout test is performed by embedding one end of the fiber into the matrix, and then the tensile force is applied to the other end of the fiber in order to pull it out from the matrix, while the force is continually monitored and recorded. The average interfacial shear strength may be calculated from the force at which debonding occurs by using Equation (3) [71,126,166,167,168,169]:

τ_app_ = F_max_/πdl (3)

where F_max_ is the maximum load measured prior to debonding of the fiber, d is the fiber diameter, and l is the fiber embedded length. 

Results related to interfacial shear strength of cement composites reinforced with modified natural fibers were previously mentioned (in the section describing the hornification thermal treatment).

#### 6.5.2. Micro-Indentation Test

The micro-indentation method is used to measure the adhesion between the fiber and the matrix. This method consists of a micro-hardness indenter, which applies a compressive force on the composite surface until a crack is observed microscopically. The micro-indentation is performed on the cross-section near the interface [170,171].

#### 6.5.3. Single Fiber Fragmentation Test

The single fiber fragmentation test is the most common measuring technique for determining micro-mechanical adhesion of long fibers. In this test, the tensile forces are transferred from the matrix to the fiber. The tensile stresses are successively applied on the fiber until the fiber fragment length is too small. The final fiber fragment length is referred to as the fiber critical length. Moreover, the final fragment length is a good indicator of the ability of the interface to transmit loads between the fiber and the matrix. The length of the fiber segments can be determined by using transmitted light microscopy and often cross-polarized light microscopy to define the stress distribution near to the fiber ends [172]. The interfacial shear strength is calculated by using Equation (4):

τ = σ_f_/2(d/IC) (4)

where τ is the interfacial shear strength of the ultimate fiber strength at the critical length, d is the fiber diameter, and IC corresponds to the fiber critical length.

## 7. Concluding Remarks and Future Trends

This paper has provided an overview on the natural fiber modification strategies to enhance fiber hydrophobicity and physico-chemical interactions at the fiber–matrix interface of cement and geopolymers composites. The common modified natural fibers used as reinforcement in cement and geopolymer matrices discussed in this review are hemp, sisal, abaca, bagasse, sweet sorghum, jute weave, fique, bamboo, eucalyptus pulps, coir, curaua, and wool fibers due to their relatively high specific strength, elastic modulus, paired with low density and cost. Pre-treatment methods aimed at improving mechanical and physical properties of the aforementioned fibers mainly included chemical agents such as sodium hydroxide (NaOH), calcium hydroxide (Ca(OH)_2_), aluminum sulfate (Al_2_(SO_4_)_3_), silane groups, 2-ethylhexanol, styrene butadiene, isocyanate, formaldehyde, and cellulose acetate biopolymer. Hornification, hydrothermal, and heat treatments were discussed as common methods used to modify natural fibers. Combination of fiber pre-treatments such as hornification prior to polymer coating displayed synergistic effects on cement composites. Furthermore, coating plant fiber with bacterial cellulose has also been investigated to reinforce cement composites.

In general, geopolymer and cement composites reinforced with these modified natural fibers exhibited promising structural performance such as interfacial strength, thermal stability, and increased mechanical properties. In this context, mainly compressive strength, flexural strength, and split tensile strength have been investigated, surprisingly with less focus on measurement of the composite fracture toughness. Additionally, the surface modification of the fibers tended to reduce water absorption capacity and setting time. Furthermore, modified fibers presented failure mechanisms related to fiber breakage, fiber pullout, and debonding from the cement and geopolymer matrices.

Analyzing the literature, it becomes apparent that more attention should be paid to the degradation mechanisms of natural fibers in geopolymer matrices, as, presumably, the typical mineralization process of plant fibers mentioned in the cement literature has not been reported in geopolymer composites. This may be expected, as mineralization is generally caused by the deposition of hydration products, likely calcium hydroxide, and geopolymer matrices are mainly composed of aluminosilicate materials. Indeed, hydration of cement matrices can be retarded by the presence of natural fibers if a high concentration of polysaccharide compounds from the fibers is released [173]. Moreover, future work needs to be performed on the durability of fiber-reinforced geopolymers for indoor and particularly outdoor applications to move away from current synthetic fiber-based composites to an alternative approach based on natural fibers as reinforcement.

On the other hand, although biological pre-treatments based on the selective degradation of lignin–hemicellulose compounds of plant fibers (by using enzymes, fungi and bacteria species) have proved to be efficient strategies to enhance the behavior of resin composites, further research still needs to be performed in the field of cement and geopolymer composites.

## Figures and Tables

**Table 1:** Qualitative analysis of the influence of chemical composition on the mechanical and physical properties of plant fibers. Based on reference [37].

Chemical Components of Plant Fibers	Parameters Correlated to Mechanical Properties				Parameters Correlated to Physical Properties		
	Tensile Strength	Specific Young’s Modulus	Failure Strain	Microfibril Angle (MFA)	Diameter	Density	Moisture Gain
Cellulose	+++	++	−	−−	+	+++	−
Hemicellulose	−	+++	−−	−−−	+	−−	++
Lignin	−−−	−−	+++	+++	−	−	++
Pectin	−	−−−	++	+++	−	+++	−−−
Wax	−	++	−−	−	−	−−−	+

Note: Symbols (+) and (−) represent, respectively, the positive and negative correlation between the chemical plant component and the mechanical and physical properties of the plant fiber.
